# Incidence and Predictors of Implantable Cardioverter-defibrillator Therapies After Generator Replacement—A Pooled Analysis of 31,640 Patients’ Data

**DOI:** 10.19102/icrm.2022.13121

**Published:** 2022-12-15

**Authors:** Dibbendhu Khanra, Subha Manivannan, Anindya Mukherjee, Saurabh Deshpande, Anunay Gupta, Wasim Rashid, Ahmed Abdalla, Peysh Patel, Deepak Padmanabhan, Indranill Basu-Ray

**Affiliations:** ^1^Liverpool Heart and Chest Hospital, Liverpool, UK; ^2^All India Institute of Medical Sciences, Rishikesh, India; ^3^NRS Medical College, Kolkata, India; ^4^Sri Jayadeva Institute of Cardiac Sciences and Research, Bengaluru, India; ^5^Vardhman Mahavir Medical College, and Safdarjung Hospital, New Delhi, India; ^6^Government Medical College, Shrinagar, India; ^7^Manchester University NHS Foundation Trust, Manchester, UK; ^8^Queen Elizabeth Hospital Birmingham, Birmingham, UK; ^9^Cardiovascular Research, Memphis Veteran Administration Hospital, Memphis, TN, USA; ^10^School of Public Health, The University of Memphis, Memphis TN, USA

**Keywords:** Generator replacement, implantable cardioverter-defibrillator, mortality, therapy

## Abstract

Among primary prevention implantable cardioverter-defibrillator (ICD) recipients, 75% do not experience any appropriate ICD therapies during their lifetime, and nearly 25% have improvements in their left ventricular ejection fraction (LVEF) during the lifespan of their first generator. The practice guidelines concerning this subgroup’s clinical need for generator replacement (GR) remain unclear. We conducted a proportional meta-analysis to determine the incidence and predictors of ICD therapies after GR and compared this to the immediate and long-term complications. A systematic review of existing literature on ICD GR was performed. Selected studies were critically appraised using the Newcastle–Ottawa scale. Outcomes data were analyzed by random-effects modeling using R (R Foundation for Statistical Computing, Vienna, Austria), and covariate analyses were conducted using the restricted maximum likelihood function. A total of 31,640 patients across 20 studies were included in the meta-analysis with a median (range) follow-up of 2.9 (1.2–8.1) years. The incidences of total therapies, appropriate shocks, and anti-tachycardia pacing post-GR were approximately 8, 4, and 5 per 100 patient-years, respectively, corresponding to 22%, 12%, and 12% of patients of the total cohort, with a high level of heterogeneity across the studies. Greater anti-arrhythmic drug use and previous shocks were associated with ICD therapies post-GR. The all-cause mortality was approximately 6 per 100 patient-years, corresponding to 17% of the cohort. Diabetes mellitus, atrial fibrillation, ischemic cardiomyopathy, and the use of digoxin were predictors of all-cause mortality in the univariate analysis; however, none of these were found to be significant predictors in the multivariate analysis. The incidences of inappropriate shocks and other procedural complications were 2 and 2 per 100 patient-years, respectively, which corresponded to 6% and 4% of the entire cohort. Patients undergoing ICD GR continue to require therapy in a significant proportion of cases without any correlation with an improvement in LVEF. Further prospective studies are necessary to risk-stratify ICD patients undergoing GR.

## Introduction

In the United States, about 800,000 people have implantable cardioverter-defibrillators (ICDs), and approximately 150,000 ICDs are implanted annually.^1^ Among primary prevention ICD recipients, 75% do not experience any appropriate ICD therapies during the lifetime of their first ICD generator.^2^ This trend could be due to goal-directed medical therapy, Multicenter Automatic Defibrillator Implantation Trial—Reduce Inappropriate Therapy (MADIT RIT)-type programming, or because half of all device recipients aged >65 years die within 5 years after device implantation.^3–5^ Nearly 25% of primary prevention ICD patients experience improvements in their left ventricular ejection fraction (EF) (LVEF) to >35% during the life of the first generator. Clinical guidance on the appropriateness of generator replacement (GR) in this cohort remains ill-defined.^6^

It has been found that patients with an LVEF of 35% are more likely to experience an appropriate ICD therapy than those with an LVEF of >35%, as was confirmed in a recent meta-analysis.^2,7^ It is undoubtedly true that the decision to replace the ICD generator requires more meticulous consideration than absolute EF in isolation, and frailty is an essential factor to be considered.^8^ Complication rates after ICD GR range from 5%–10% and tend to be higher in concert with lead upgrades, multiple replacements, and patient frailty.^9–11^ Of note, experiencing a major complication during ICD implantation is associated with a significantly higher risk of mortality for up to 6 months after the procedure.^12^ Moreover, although ICDs reduce arrhythmic death, 25% of patients die after their first ICD generator replacement without ever experiencing an appropriate ICD therapy, and improvement in EF has not been proven as a predictor of all-cause mortality, thus reiterating that ICDs may not reduce non-arrhythmic death.^2^ Therefore, we conducted a proportional meta-analysis to determine the incidence and predictors of ICD therapies after GR and compared the immediate and long-term complications. The need for ICD GR, especially in primary prevention cohorts with no previous therapies and improved LVEFs, has been debated. This study attempts to answer this question, which has important implications as electrophysiologists commonly face this dilemma in their daily practice.

## Methods

### Search strategy

A systematic review of existing literature on ICD GR in primary prevention patients was performed to search for studies published prior to December 2021. Two physician-reviewers queried PubMed, the Web of Science, and the Cochrane Library of Controlled Trials (CENTRAL) databases for published literature, using keywords such as “implantable cardioverter-defibrillator,” “generator replacement,” “primary prevention,” and “therapies” alone and in combination. Additional literature was sought by searching the references of eligible articles. A third reviewer resolved any discrepancies.

### Study selection

**Figure 1:**
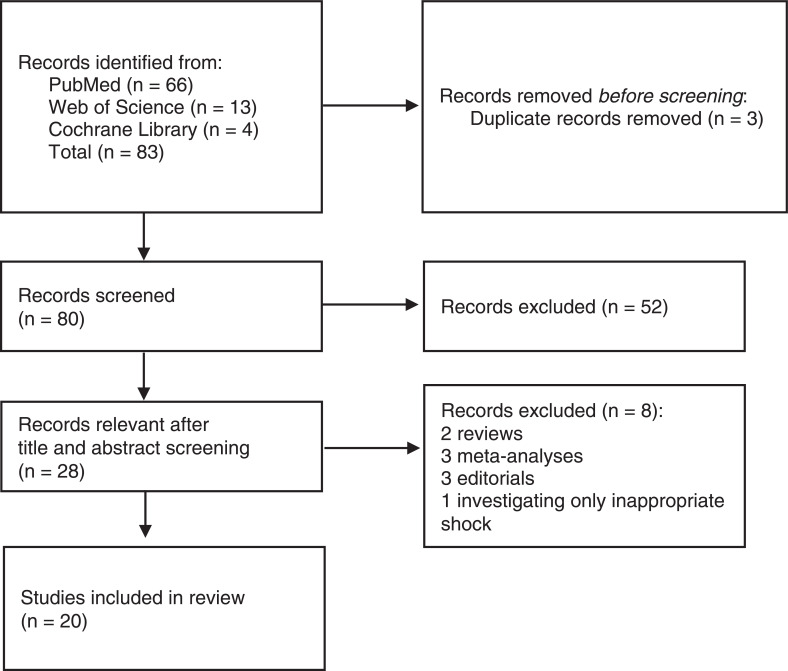
Preferred Reporting Instrument for Systematic Reviews and Meta-analysis flow diagram.

For the meta-analysis, we selected observational studies and registries that reported data on ICD GR in primary and secondary prevention patients. Case reports, case series, editorials, and review articles were excluded. Only studies on transvenous ICDs were included. Details are given in the Preferred Reporting Instrument for Systematic Reviews and Meta-analysis flow diagram **(Figure 1)**.^13^ Studies were critically appraised using the Newcastle–Ottawa scale **(Table S1)**.^14^ This meta-analysis has been registered in PROSPERO (identifier: CRD42021288546).

### Data extraction

Baseline characteristics included the type of study, total number of patients, follow-up duration, year of publication, sex ratio, age, diabetes, hypertension, chronic kidney disease (CKD), body mass index, atrial fibrillation (AF), indication (primary or secondary), previous therapies (anti-tachycardia pacing [ATP] therapies or shocks), LVEF, improvement in LVEF, biventricular device, ischemic cardiomyopathy (ICM), New York Heart Association (NYHA) class, QRS duration, heart failure (HF) medications, and anti-arrhythmic drugs (AADs). In addition, outcomes such as procedural complications, therapies (shocks and ATPs), inappropriate shocks, and all-cause mortalities were also extracted from the individual studies.

### Outcome

The primary outcomes of the meta-analysis were the incidence rates and determinants of ICD therapies and all-cause mortality, while secondary outcomes were incidence rates of ICD shocks, ATP, inappropriate shocks, and procedure-related complications. Based on the available data, attempts were made to perform covariate analyses of all the primary and secondary outcomes.

### Data analysis

To pool the incidence of outcomes across all the selected studies, the R software (RStudio: Integrated Development for R; RStudio, Inc., Boston, MA, USA) using the “metarate” function was used to derive the incidence rate ratio in 100 patient-years. A random-effects model was used to circumvent heterogeneity so that no particular study was given a higher weightage. The outcome data were transformed by the logarithmic method to increase their statistical value and back-transformed to proportions using the “escal” function in “metafor.”^15^ Forest plots were drawn with proportional measures and 95% confidence intervals (CIs) using a random-effects model. Study heterogeneity was expressed as *I*^2^. Influencing and outlier studies were looked for, and sensitivity analyses were conducted. Univariate and multivariate logarithmic covariate analyses of the outcomes were performed using the restricted maximum likelihood (REML) function. *R*^2^ was used to estimate the heterogeneity accounted for, and a bubble plot was drawn to visualize the moderator effects.

## Results

A total of 31,640 patients from 20 studies (14 retrospective and 6 prospective registries) were included in our meta-analysis **(Figure 1)** with a mean ± standard deviation age of 65.6 ± 4.7 years (mean age range, 57–76 years) and follow-up period of 3.1 ± 1.5 years.^2,6,10,11,16–31^ The critical appraisal performed using the Newcastle–Ottawa scale revealed that the quality of studies was good **(Table S1)**.

### Baseline data

Eighty-one percent of patients in the meta-analysis cohort were male. Most of the studies defined an improvement of LVEF as LVEF ≥ 35%; however, House et al.^19^ defined it as LVEF ≥ 50%, and Kini et al.^6^ and Sebag et al.^17^ both defined it as LVEF ≥ 40%. The baseline LVEF was 26.7% ± 3.4% in the entire cohort, with 58.9% of patients overall having ICM, and proportions of patients with NYHA class 3 or 4 symptoms ranged from 14.6%–76.6% across the studies. The common comorbidities included hypertension (59.5%), diabetes mellitus (DM) (31.1%), CKD (21.8%), and AF (36.5%). Overall, 45.6% of patients received cardiac resynchronization therapy with defibrillation (CRT-D), and 78.4%, 84.4%, 39.6%, and 27.9% of patients were on angiotensin-converting enzyme inhibitors/angiotensin II receptor blockers (ACEI/ARBs), β-blockers, mineralocorticoid inhibitors, and digoxin, respectively. AADs were prescribed to 20.7% of patients. These ICDs were programmed in multiple zones with therapy advised for fast and sustained ventricular arrhythmias starting with ATP followed by shock. Only Witt et al.^26^ and Narducci et al.^27^ reported secondary prevention ICD replacements in 51.5% and 7.86% of patients, respectively; the rest of the studies reported on patients with primary prevention ICDs. In 3 studies,^2,18,19^ patients without any ICD therapies in the first life of the generator were selected. In all the other studies, nearly 1/3 of patients had some form of therapy **(Table 1)**.

### Implantable cardioverter-defibrillator therapies

**Figure 2:**
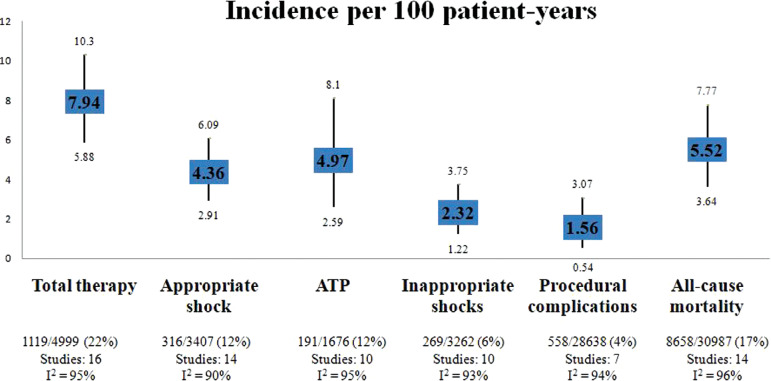
Incidence rate ratio of outcomes. *Abbreviation*: ATP, anti-tachycardia pacing.

Total therapies among patients after GR numbered 7.94 (95% CI, 5.88–10.3; *I*^2^ = 95%) per 100 patient-years; ATP predominated at 4.97 (95% CI, 2.59–8.1; *I*^2^ = 95%) per 100 patient-years, followed by appropriate shocks (4.36 per 100 patient-years [95% CI, 2.91–6.09]; *I*^2^ = 90%) **(Figure 2)**.

### Total therapy

**Figure 3:**
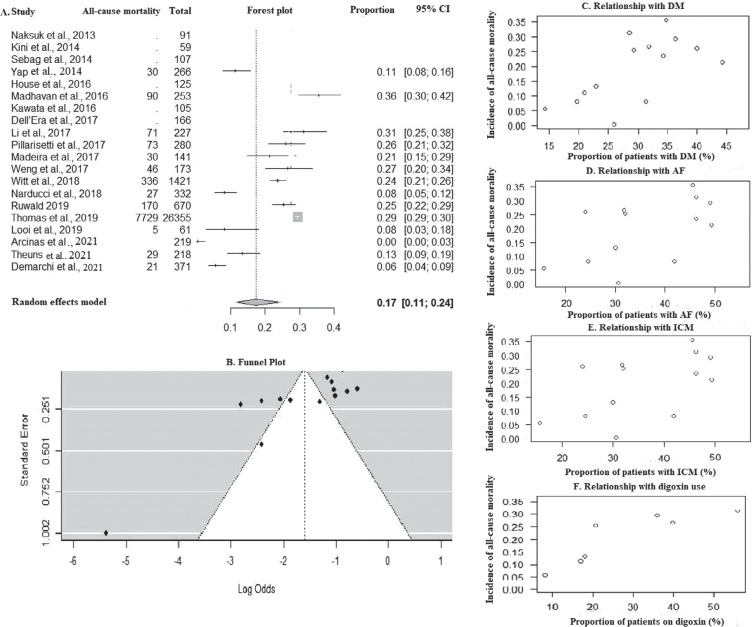
**A:** Forest plot of implantable cardioverter-defibrillator therapies. **B:** Funnel plot. Bubble plots show **C:** relationship with AAD, **D:** relationship with previous shocks, and **E:** relationship with previous ATPs. *Abbreviations*: AAD, antiarrhythmic drug; ATP, anti-tachycardia pacing; CI, confidence interval; ICD, implantable cardioverter-defibrillator.

ICD therapies were delivered to 22% of the entire cohort **(Figure 3A)**. The study by Witt et al. reported 51.5% of patients had secondary prevention ICDs; however, excluding this study, the incidence of ICD therapies remained at 21%. Incidence rates of ICD therapies in retrospective and prospective studies were 7.7 and 8.5 events per 100 patient-years, respectively. The funnel plot did not reveal any significant asymmetry (Kendall’s τ = 0.0588; *P* = .7765) **(Figure 3B**). The proportion of total ICD therapies was significantly higher with the use of AADs (effect size [ES] [95% CI], 0.03 [0.01–0.06]; *P* = .007; *R*^2^ = 41%) **(Figure 3C)**, shocks (ES [95% CI], 0.14 [0.09–0.19]; *P* < .0001; *R*^2^ = 100%) **(Figure 3D)**, and ATP (ES [95% CI], 0.06 [0.02–0.10]; *P* = .002; *R*^2^ = 85%) **(Figure 3E)** from the device in its first generator life. ICD therapies did not significantly correlate with LVEF or follow-up duration across the studies **(Table 2)**. In the multivariate analysis, the use of AAD and previous shocks was found to be associated with higher ICD therapies following GR **(Table 2)**.

### Implantable cardioverter-defibrillator shocks

**Figure S1:**
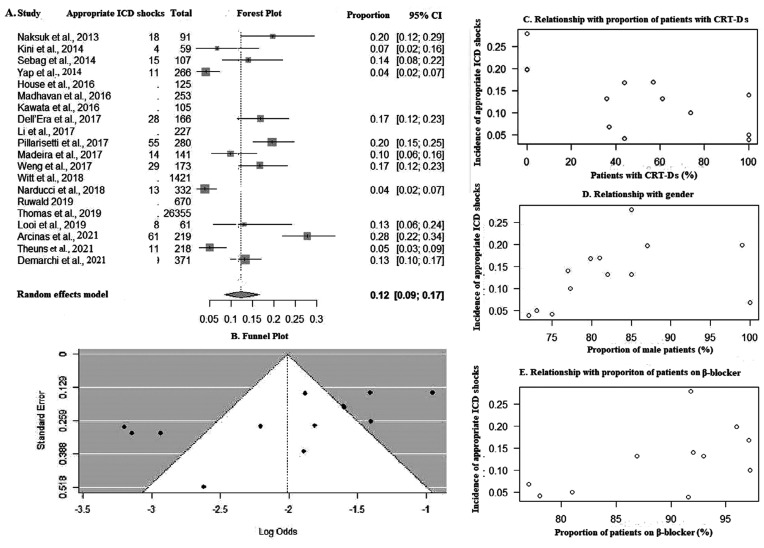
**A:** Forest plot of ICD shocks. **B:** Funnel plot. Bubble plots showing relationship with **C:** proportion of patients with cardiac resynchronization therapy defibrillator, **D:** gender, and **E:** proportion of patients on β-blocker. *Abbreviations:* CI, confidence interval; CRT-D, cardiac resynchronization therapy-defibrillator; ICD, implantable cardioverter-defibrillator.

Twelve percent of patients of the entire cohort received ICD shocks in our pooled analysis **(Figure S1A)**. A funnel plot is shown in **Figure S1B** (Kendall’s τ = −0.5128; *P* = .0150). Bivariate analysis revealed a significantly lower number of shocks in patients with CRT-Ds (ES [95% CI], −0.01 [−0.02 to −0.004]; *P* = .002; *R*^2^ = 58%) **(Figure S1C)** and a significantly higher number of shocks in men (ES [95% CI], 0.04 [0.0007–0.08]; *P* = .04; *R*^2^ = 34%) **(Figure S1D)**. The use of β-blockers was found to have a positive correlation with the proportion of ICD shocks in the bivariate analysis (ES [95% CI], 0.06 [0.009–0.12]; *P* = .02) and multivariate analysis (ES [95% CI], 0.05 [0.01–0.1]; *P* = .008) **(Figure S1E)**. The incidence of shocks was not found to be affected by LVEF (ES [95% CI], −0.01 [−0.12 to 0.08]; *P* = .42) or previous shocks from the device (ES [95% CI], 0.02 [−0.02 to 0.07]; *P* = .37) **(Table S2)**.

### Anti-tachycardia pacing

**Figure S2:**
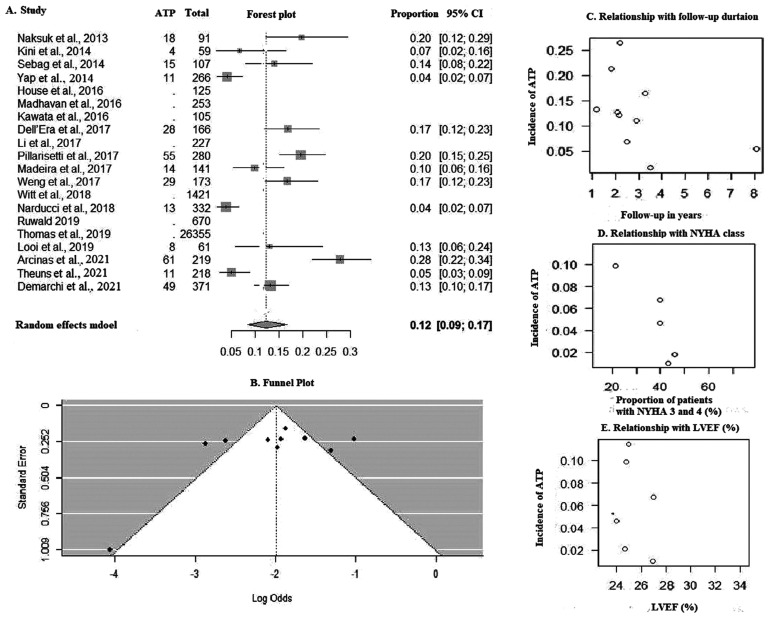
**A:** Forest plot of anti-tachycardia pacing. **B:** Funnel plot. Bubble plot showing relationship with **C:** follow-up duration, **D:** New York Heart Association class, and **E:** left ventricular ejection fraction. *Abbreviations:* AF, atrial fibrillation; CI, confidence interval; DM, diabetes mellitus; ICM, ischemic cardiomyopathy.

Twelve percent of patients in the entire cohort received ATPs in our pooled analysis **(Figure S2A)**. There was no evidence of publication bias **(Figure S2B)** in the funnel plot (Kendall’s τ = −0.2889; *P* = .2912). Bivariate analysis showed a significantly higher ATP with shorter follow-up (ES [95% CI], −0.18 [−0.35 to −0.007]; *P* = .04; *R*^2^ = 34%) **(Figure S2C)** and in NYHA class 3 or 4 patients (ES [95% CI], −0.03 [−0.06 to −0.003]; *P* = .002; *R*^2^ = 52%), but these findings were not found to be significant in the multivariate analysis **(Figure S2D)**. The incidence of ATP was not found to be affected by LVEF (ES [95% CI], 0.06 [−0.02 to 0.15]; *P* = .14) **(Figure S2E)**. The results of bivariate and multivariate analyses are presented in **Table S3**.

### All-cause mortality

**Figure 4:**
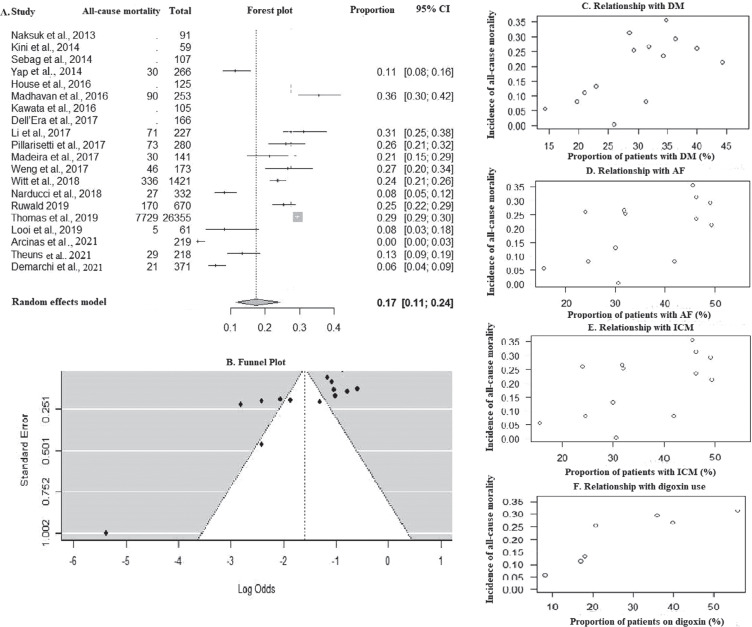
**A:** Forest plot of all-cause mortality. **B:** Funnel plot. Bubble plot showing relationship with **C:** diabetes mellitus, **D:** atrial fibrillation, **E:** ischemic cardiomyopathy, and **F:** digoxin use. *Abbreviations*: AF, atrial fibrillation; CI, confidence interval; DM, diabetes mellitus; ICM, ischemic cardiomyopathy.

The all-cause mortality was found to be 5.52 (95% CI, 3.64–7.77; *I*^2^ = 96%) per 100 patient-years **(Figure 2)**. The proportion of all-cause mortality was 17% in the total cohort **(Figure 4A)**. A funnel plot is depicted in **Figure 4B** (Kendall’s τ = −0.4505; *P* = .026). During bivariate analysis, DM (ES [95% CI], 0.06 [0.002–0.02]; *P* = .002; *R*^2^ = 50%) **(Figure 4C)**, concurrent AF (ES [95% CI], 0.04 [0.003–0.08]; *P* = .03; *R*^2^ = 28%) **(Figure 4D)**, ICM (ES [95% CI], 0.04 [0.02–0.05]; *P* < .0001; *R*^2^ = 77%) **(Figure 4E)**, and digoxin use (ES [95% CI], 0.03 [0.01–0.05]; *P* = .001; *R*^2^ = 64%) **(Figure 4F)** were found to be associated with higher all-cause mortality rates, but they did not independently affect all-cause mortality in the multivariate analysis **(Table 3)**. Follow-up duration (ES [95% CI], 0.18 [−0.08 to 0.45]; *P* = .18) and improvements in LVEF (ES [95% CI], −0.01 [−0.06 to 0.03]; *P* = .61) did not affect mortality **(Table 3)**.

### Inappropriate shocks

**Figure S3:**
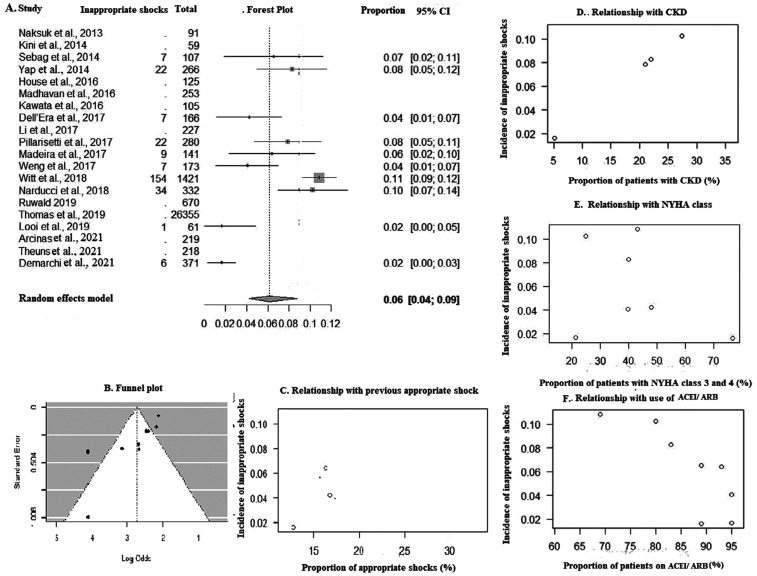
**A:** Forest plot of inappropriate therapies. **B:** Funnel plot. Bubble plot showing relationship with **C:** appropriate shocks, **D:** chronic kidney disease, **E:** New York Heart Association class, and **F:** use of angiotensin-converting enzyme inhibitor/angiotensin receptor blocker therapy. *Abbreviations*: ACEI, angiotensin-converting enzyme inhibitor; ARB, angiotensin receptor blocker; CI, confidence interval; CKD, chronic kidney disease; NYHA, New York Heart Association.

The incidence of inappropriate shocks was 2.32 (95% CI, 1.22–3.75; *I*^2^ = 93%) per 100 patient-years **(Figure 2)**, affecting 6% of patients of the entire cohort **(Figure S3A)**. A funnel plot is depicted in **Table S3B** (Kendall’s τ = −0.6444; *P* = .0091) **(Figure S3B)**. The bivariate analysis showed that inappropriate shocks were more frequent in subjects who received a higher proportion of appropriate shocks from the device during the first life of the battery (ES [95% CI], 0.31 [0.02–0.59]; *P* = .03; *R*^2^ = 88%) **(Figure S3C)** and in CKD patients (ES [95% CI], 0.08 [0.04–0.12]; *P* < .0001; *R*^2^ = 100%) **(Figure S3D)** and less frequent in patients with NYHA class 3–4 (ES [95% CI], −0.02 [−0.05 to −0.0006]; *P* = .04; *R*^2^ = 52%) **(Figure S3E)** and in ACEI/ARB users (ES [95% CI], −0.04 [−0.08 to −0.007]; *P* = .02; *R*^2^ = 0%) **(Figure S3F)**, but none of these findings were significant in the multivariate analysis **(Table S4)**.

### Procedure-related complications

**Figure S4:**
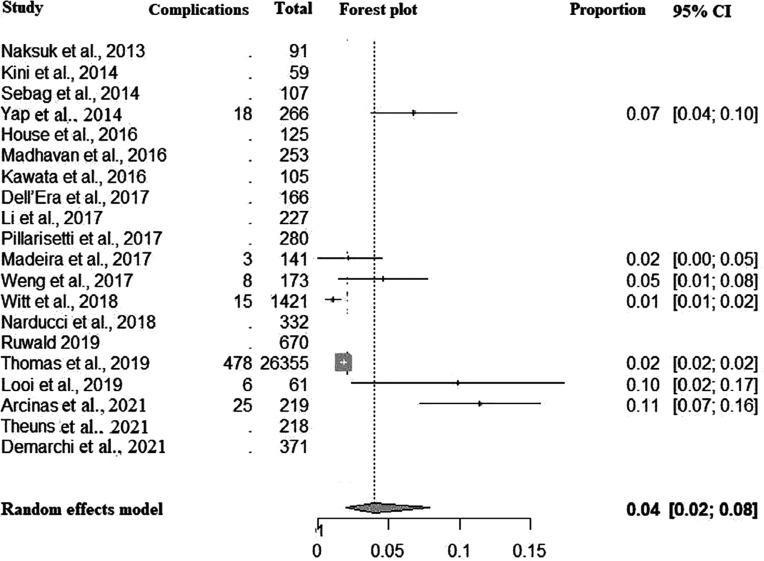
Forest plot of procedure-related complications. *Abbreviation:* CI, confidence interval.

Procedural-related complications totaled 1.56 (95% CI, 0.54–3.07; *I*^2^ = 90%) per 100 patient-years **(Figure 2)**, which corresponded to 4% of patients in the entire cohort **(Figure S4)**. Most complications were lead fracture (4.7%), followed by pocket/site infection (2.5%) and pocket hematoma (1.1%) **(Table S5)**.

## Discussion

In this proportional meta-analysis of 31,640 patients across 20 studies who had received ICDs predominantly for primary prevention, the incidence of total therapies post-GR was 8 per 100 patient-years, whereas the incidence of all-cause mortality was 5.52 per 100 patient-years in the entire cohort. Also, the incidence rates of inappropriate shock and other procedural complications were low at 2.32 and 1.56 per 100 patient-years, respectively. The use of AADs was independently associated with more frequent ICD therapies, and previous ICD shocks but not ATPs were an independent predictor of ICD therapies. Finally, LVEF did not affect total therapies from ICD or all-cause mortality.

### Implantable cardioverter-defibrillator therapies

Our meta-analysis showed that using AADs independently increases ICD therapies in primary prevention ICD patients undergoing GR. Patients receiving AADs may experience drug–device interactions, which lead to pro-arrhythmic effects that alter the tachycardia cycle length and/or change the QT interval, which may lead to more frequent ICD therapies.^32,33^ Although this may be true due to the risk of pro-arrhythmia with these medications, it is equally plausible that the use of anti-arrhythmic medications is a marker of previous arrhythmias and, therefore, would be associated with an increased risk for future arrhythmias. Our meta-analysis revealed that ICD shocks in the first life of the generator predict overall ICD therapies following GR, which resonated with the findings from Arcinas et al.’s study.^11^

In our meta-analysis, patients with appropriate shocks were associated with the use of β-blockers probably because β-blockers are more likely to be used in patients with previous shocks or those at high risk for shock and primary prevention patients with HF with reduced ejection fraction. The Optimal Pharmacological Therapy in Cardioverter-defibrillator Patients (OPTIC) trial evaluated the effect of amiodarone, β-blockers, sotalol, and the combination of amiodarone and β-blockers on ICD shocks and showed that shocks were lower in the combination amiodarone–β-blocker and sotalol arms than in the β-blockers arm.^34^ The addition of β-blockers to ICD therapy has been found to reduce HF-related hospitalization and mortality rates.^35^

LVEF is currently our best risk-stratification tool when planning for primary prevention ICD implantation. However, our meta-analysis shows that the absolute improvement of LVEF did not affect total therapies, appropriate shocks, or ATP in patients who had undergone GR. Therefore, we propose that LVEF in isolation should not be considered the sole criterion for deciding on GR in these patients. Furthermore, though LVEF might improve the pro-arrhythmic substrate in the form of scars, initiating and sustaining arrhythmogenic foci may linger, thus prompting the need for an ICD to prevent sudden cardiac death.

### Mortality

In this meta-analysis, DM, an ischemic cause of low LVEF, concurrent AF, and greater use of digoxin were associated with higher all-cause mortality, but they did not independently affect all-cause mortality. LVEF, or an improvement in LVEF, also did not affect mortality. The role of digoxin in HF has been dubious, and studies have shown that digoxin can be related to increased mortality in patients with HF.^36^ Merchant et al. recommended that the decision for GR should be made while keeping in mind the relative risks of arrhythmic and non-arrhythmic deaths. It is mandatory to comprehend that, since initial implant patients undergoing GR are older and may have a plethora of existing and or newer comorbidities, they would ostensibly have a shorter lifespan, which may limit the benefit of ICD therapy. Moreover, the probability of additional complications, including infections, cannot be discounted.^37^ Thus, quality of life should be considered, and the patient should have a life expectancy of ≥1 year and a reasonable quality of life to justify GR.^38^ Lastly, Pillarisetti et al. pointed out that, in the study by Kini et al.,^6^ the rate of ICD therapies was 1.4% per year despite LVEF improvement, which was much higher than the 0.1% risk of sudden cardiac death in the general population. Thus, if the general population was taken as a control group, the absolute risk reduction would be 1.3%, with the number needed to treat being 76 patients.^39^

### Complications (inappropriate shocks and procedural issues)

We have shown that the risks of procedure-related complications and inappropriate shocks are very low (6% and 4%, respectively). Rates of procedural complications in ICD GR cases can be notoriously higher than during the first implant owing to patient frailty, and complications tend to be more common also in cases of lead upgrades and multiple GRs. Looi et al.^10^ reported the highest complication rate (10%), possibly due to lead fractures in 6% of patients and upgrades to CRT in nearly 35% of the patients, whereas complications were very low (2%) in the large registry by Thomas et al.^29^ Lead fracture as a risk for mortality was likely reflective of the Fidelis (Medtronic, Minneapolis, MN, USA) and Riata (Abbott, Chicago, IL, USA) era. Although the rate of ICD failures from lead fractures is much less in the contemporary world, cardiovascular implantable electronic device infections are likely to predict mortality. However, only Arcinas et al. reported 1.4% lead infections in their ICD cohorts.^11^ It is also known from the Multicenter Electrophysiologic Device Infection Cohort (MEDIC) trial that the infection risk for ICD replacement increases exponentially with each pocket re-entry.^40^ In our meta-analysis, inappropriate shocks were found to be more common in CKD patients, which reflected the finding by Bansal et al., which showed that patients with CKD have a higher probability of having inappropriate shocks, probably owing to a higher incidence of hyperkalemia and AF in this population.^41^ The lower incidence of inappropriate shocks with ACE/ARB therapy might be related to the improving overall HF status.

### Limitations

The present work of meta-analysis is based on pooled data across the selected studies. However, we did not have access to individual patient data and thus had to perform the statistical analysis based only on the published data. A huge male bias was observed in the pooled patients’ data. The dataset was grossly heterogeneous, and we did not perform a comparative meta-analysis or subgroup analysis (between patients with improved and not improved LVEFs) due to the lack of separate outcome data in many studies. Some studies did not specify the indications for ICDs (primary or secondary). Device programming was not specified in detail and may have variations across the studies. Some studies did not mention the incidences of previous therapies (such as shocks or ATPs). Most of the studies did not enumerate complications. Insufficient data restricted our ability to perform covariate analysis for complications. Objective assessments of funnel plots depicting incidence rates of appropriate and inappropriate shocks and all-cause mortality were asymmetric, suggesting very heterogeneous data rather than publication bias in the context of proportional meta-analysis.^15^ The findings of the covariate analysis of the outcomes in the meta-analysis need further assessment in prospective registries and may not be replicated in clinical practice.

## Conclusion

The need for GR of ICDs, especially in primary prevention cohorts, with no previous therapies and improved LVEFs, has been questioned. We contend that ensuring informed consent from and sharing decision-making with patients are essential. Our findings portend a significant residual risk of sudden cardiac death in this cohort irrespective of the improvement in LVEF. In our large cohort, the use of AADs was associated with an increased risk for appropriate ICD therapies. The pathophysiological etiology of this phenomenon remains to be determined. It is also essential to comprehend that, while GR of ICDs prevents arrhythmic death, it has no bearing on all-cause mortality.

Furthermore, the risk of complications associated with GR is minimal in most circumstances. The benefit offered by GR thus squarely outweighs its risk, though not in all cases. Decision-making should thus be individualized, catering to the individual patient-associated mortality and morbidity. A consensus thus arrived at and discussed with the patient will enable an understanding of the risk–benefit ratio for a GR with great precision. A detailed prospective analysis is mandated to substantiate our findings. It is also evident that a better comprehension of the predictors of therapies needs to be defined, which may help us decide on GR.

## Figures and Tables

**Table 1:** Baseline Parameters Across the Studies

Study	n	FU (months) Mean, SD	Male (%)	Age (years) Mean, SD	DM (%)	HTN (%)	CKD (%)	AF (%)	LVEF (%) Mean, SD	Previous ICD Therapies (%)	Improved LVEFa (%)	CRT-D (%)	ICM (%)	NYHA 3–4 (%)	ACEI/ARB (%)	BB (%)	MA (%)	Digoxin (%)	AAD (%)
Madhavan et al., 2016^2^	253	39.6 (21.6–63.6)^b^	82	68.3, 12.7	34.8	75.9		45.5	32.2, 12.4	No previous therapy	28	0	81.9	14.6	84.5	93.1			
Kini et al., 2014^6^	152		100	65, 10	46	82	29	24	23, 6		26	37	69		86	77			13
Looi et al., 2019^10^	61	21.6, 18	81.9	58.2, 11.4	19.7	22.9		24.6	24.8, 5.2	Total 29.5		36.1	31.7	21.3	95.1	86.9			24.6
Arcinas et al., 2021^11^	219	27 (17–43)^b^	85	67, 12	26.00	52		31	24 (20–29)^b^	Shock 33.3, ATP 59.2		0	63.4		79	92	39.7		29.20
Naksuk et al., 2013^16^	91	26.4, 19.2	99	70, 11	47.5	78.2			34, 10.5	Shock 23, ATP 32.9	27	0	76	18.5	78	96	14		
Sebag et al., 2014^17^	107	26.4, 14.4	77	65, 11	21	28		22	26, 7		37	100	46		89	92	42		17
Yap et al., 2014^18^	266	30, 24	75	57, 12	21		22		27, 10	No previous therapy		44	59	40	83	78		17	15
House et al., 2016^19^	125	25, 18	74	64,14	29.6	73.6		53.6	25, 7	No previous therapy	24	58	72		61.1	91.2			18.04
Kawata et al., 2016^20^	168	41.2, 26.5	75	66.3, 12.0					26.9, 7.1	Total 37.5	47	0	63.1		85.7	95.8			23.2
Dell’Era et al., 2017^21^	166	25, 23	81	66, 10				55.4	33, 11	Total 28.9, shock 16.8	38.5	57	54	48					16
Li et al., 2017^22^	227		79.3	66.6, 11.4	28.6	35.1		46.3	23.6, 6.8	Total 29	48	100	65.5	70	87.7	88.6	27.2	56	
Pillarisetti et al., 2017^23^	280	61, 27	87	64, 12	40	76	21	24	24, 7.5	Total 25.2	30	0	72						8
Madeira et al., 2017^24^	141	39.3, 18.5	77.3	60.9, 10.7	44.3	60.4		49.3	24.7, 6.2	Total 22.7, shock 16.3, ATP 13.5	36	73.8	50.4		93.6	97.2	66.7		31.2
Weng et al., 2017^25^	173	34.8, 27.6	79.8	67.0, 10.5	31.8	45.1		31.8	24, 6.0	Total 42.2	24.2	43.9	68.2	39.8	94.8	97.1	31.8	39.9	
Witt et al., 2018^26,c^	1,421	32.4, 31.2	81	69.6, 12.1	34.3	63.1		46.3	26.9, 13.3	Total 33.1	38.9	0	68.1	43.1	69.6	83			31.2
Narducci et al., 2018^27,c^	332		72	71	31.3	64.8	27.4	41.9	34.3, 10	Total 31.3	34.5	100	52.4	25	79.8	91.6			20.8
Ruwald et al., 2019^28^	670	24, 19.2	79.1	69.3, 9.7	29.3		11.8	32	24.4, 7.2	Total 29.4		50	76.9		85.2	92.5		20.6	15.5
Thomas et al., 2019^29^	26,355		76.7	76, 6.6	36.3	76.5	2.8^d^	49.1			24.7		76.4	45.8	69.7	85		36	
Theuns et al., 2021^30^	218	50.4 (24–81.6)^b^	73	65 (58–71)^b^	23		36	30	25, 6	Shock 12		100	43	72	96	81	41	18	
Demarchi et al., 2021^31^	371	34 (18–55)^b^	82	58 (49–67)^b^	14.3		5	15.6	28 (23–35)^b^	Shock 12.8, ATP 12.1		61	41.8	76.6	89	93	54	8	27

*Abbreviations:* AAD, anti-arrhythmic drug; ACEI/ARB, angiotensin-converting enzyme inhibitor/angiotensin receptor blocker; AF, atrial fibrillation; ATP, anti-tachycardia pacing; BB, β-blocker; BMI, body mass index; CKD, chronic kidney disease; CRT-D, cardiac resynchronization therapy defibrillator; DM, diabetes mellitus; FU, follow-up; HTN, hypertension; ICD, implantable cardioverter-defibrillator; ICM, ischemic cardiomyopathy; LVEF, left ventricular ejection fraction; MA, mineralocorticoid antagonist; NYHA, New York Heart Association; SD, standard deviation. ^a^House et al.^19^ defined LVEF as EF ≥ 50% and Kini et al.^6^ and Sebag et al.^17^ defined it as LVEF ≥ 40%. ^b^Median (interquartile range). ^c^Witt et al.^26^: secondary prevention, 46%; Narducci et al.^27^: secondary prevention, 7.86%. ^d^Patients on hemodialysis.

**Table 2:** Covariate Analysis of Total Appropriate Implantable Cardioverter-defibrillator Therapies (ie, Shocks and Anti-tachycardia Pacing)

	Univariate				Multivariate			
	ES (95% CI)	*Z* Value	*P* Value	*R*^2^ (%)	ES (95% CI)	*Z* Value	*P* Value	*R*^2^ (%)
Sample size	0.01 (−0.007 to 0.03)	1.21	.22	11.36				
Study design	**0.50 (0.07–0.94)**	**2.28**	**.02**	**37.25**	0.12 (−0.38 to 0.63)	0.47	.63	36.69
Follow-up (years)	−0.05 (−0.22 to 0.12)	−0.58	.56	0				
Mean age in years	0.02 (−0.02 to 0.08)	0.96	.33	0.08				
Male	0.02 (−0.004 to 0.06)	1.72	.08	22.35				
DM	0.01 (−0.01 to 0.04)	0.97	.33	4.05				
HTN	−0.002 (−0.01 to 0.01)	−0.34	.73	0				
CKD	−0.01 (−0.02 to 0.007)	−1.13	.25	0				
Mean BMI (kg/m^2^)	0.11 (−0.25 to 0.48)	0.6	.54	0				
Mean QRS (ms)	−0.02 (−0.06 to 0.02)	−0.95	.34	0				
AF	0.01 (−0.006 to 0.02)	1.26	.2	5.57				
ICM	0.001 (−0.01 to 0.02)	0.16	.87	0				
NYHA 3,4	−0.01 (−0.02 to 0.005)	−1.37	.16	8.85				
Patients with improved LVEF	−0.006 (−0.02 to 0.01)	−0.52	.6	0				
Mean LVEF (%)	0.03 (−0.03 to 0.10)	0.98	.32	0				
CRT-D	−0.005 (−0.01 to 0.001)	−1.62	.1	13.42				
ACEI/ARB	−0.008 (−0.05 to 0.03)	−0.41	.68	0				
BB	0.03 (−0.007 to 0.08)	1.65	.09	7.94				
MA	−0.01 (−0.03 to 0.005)	−1.48	.13	18.88				
Digoxin	0.01 (−0.007 to 0.03)	0.01	.22	11.36				
AAD	**0.03 (0.01**–**0.06)**	**2.69**	**.007**	**40.94**	**0.03 (0.001**–**0.06)**	**2.02**	**.04**	
Previous shocks	**0.14 (0.09**–**0.19)**	**5.77**	**<.0001**	**100**	**0.07 (0.005**–**0.10)**	**4.65**	**.02**	
Previous ATP	**0.06 (0.02**–**0.10)**	**3.01**	**.002**	**85.36**	0.01 (−0.002 to 0.10)	1.27	.65	

*Abbreviations:* AAD, anti-arrhythmic drug; ACEI/ARB, angiotensin-converting enzyme inhibitor/angiotensin receptor blocker; AF, atrial fibrillation; ATP, anti-tachycardia pacing; BB, β-blocker; BMI, body mass index; CI, confidence interval; CKD, chronic kidney disease; CRT-D, cardiac resynchronization therapy defibrillator; DM, diabetes mellitus; ES, effect size; HTN, hypertension; ICD, implantable cardioverter-defibrillator; ICM, ischemic cardiomyopathy; LVEF, left ventricular ejection fraction; MA, mineralocorticoid antagonist; NYHA, New York Heart Association. Bolded values are statistically significant (*P* < 0.05).

**Table 3:** Covariate Analysis of All-cause Mortality

	Univariate				Multivariate			
	ES (95% CI)	*Z* Value	*P* Value	*R*^2^ (%)	ES (95% CI)	*Z* Value	*P* Value	*R*^2^ (%)
Sample size	0.00003 (−0.00003 to 0.00009)	0.91	.36	0				
Study design	0.08 (−0.57 to 0.73)	0.24	.8	0				
Follow-up (years)	0.18 (−0.08 to 0.45)	1.33	.18	5.47				
Mean age in years	0.06 (−0.07 to 0.14)	1.76	.07	23.78				
Male gender	−0.008 (−0.12 to 0.10)	−0.13	.88	0				
DM	**0.06 (0.002**–**0.02)**	**3.04**	**.002**	**49.8**	0.03 (−0.09 to 0.16)	0.55	.57	79.1
HTN	0.01 (−0.01 to 0.05)	0.01	.94	0				
CKD	0.003 (−0.05 to 0.06)	0.16	.87	0				
Mean BMI (kg/m^2^)	0.59 (−0.18 to 1.37)	1.49	.13	29.7				
Mean QRS (ms)	−0.03 (−0.07 to 0.006)	−1.63	.1	27.4				
AF	**0.04 (0.003**–**0.08)**	**2.11**	**.03**	**28.3**	0.001 (−0.06 to 0.06)	0.04	.96	
ICM	**0.04 (0.02**–**0.05)**	**4.68**	**<.0001**	**77.3**	0.01 (−0.02 to 0.06)	0.69	.48	
NYHA 3, 4	−0.006 (−0.03 to 0.01)	−0.46	.64	0				
Patients with improved LVEF	−0.01 (−0.06 to 0.03)	−0.5	.61	0				
Mean LVEF (%)	−0.05 (−0.20 to 0.09)	−0.7	.47	0				
CRT-D	−0.001 (−0.01 to 0.01)	−0.23	.81	0				
ACEI/ARB	−0.008 (−0.06 to 0.05)	−0.28	.78	0				
BB	0.009 (−0.08 to 0.09)	0.2	.84	0				
MA	−0.01 (−0.10 to 0.08)	−0.29	.77	0				
Digoxin	**0.03 (0.01 to 0.05)**	**3.23**	**.001**	**64.1**	0.01 (−0.01 to 0.04)	1.02	.3	
AAD	−0.04 (−0.12 to 0.04)	−0.95	.33	0				
Previous shocks	−0.15 (−0.30 to 0.0001)	−1.95	.05	51.7				
Previous ATP	0.05 (−0.14 to 0.001)	−1.92	.05	63.6				

*Abbreviations:* AAD, anti-arrhythmic drug; ACEI/ARB, angiotensin-converting enzyme inhibitor/angiotensin receptor blocker; AF, atrial fibrillation; ATP, anti-tachycardia pacing; BB, β-blocker; BMI, body mass index; CI, confidence interval; CKD, chronic kidney disease; CRT-D, cardiac resynchronization therapy-defibrillator; DM, diabetes mellitus; ES, effect size; HTN, hypertension; ICM, ischemic cardiomyopathy; LVEF, left ventricular ejection fraction; MA, mineralocorticoid antagonist; NYHA, New York Heart Association. Bolded values are statistically significant (*P* < 0.05).

**Table S1:** Critical Appraisal of the Studies According to the Newcastle–Ottawa Scale

Study	Study Design	Selection	Comparability	Outcome	AHRQ Score
Madhavan et al., 2016^2^	Retrospective cohort	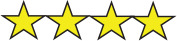	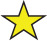	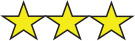	Good
Kini et al., 2014^6^	Retrospective cohort	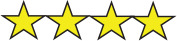	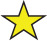	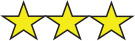	Good
Looi et al., 2019^10^	Retrospective cohort	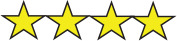	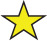	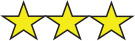	Good
Arcinas et al., 2021^11^	Retrospective cohort	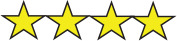	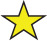	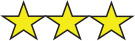	Good
Naksuk et al., 2013^16^	Retrospective cohort	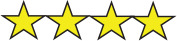	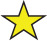	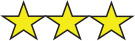	Good
Sebag et al., 2014^17^	Prospective cohort	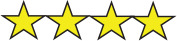	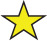	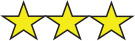	Good
Yap et al., 2014^18^	Prospective cohort	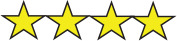	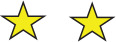	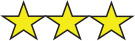	Good
House et al., 2016^19^	Retrospective cohort	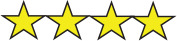	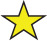	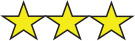	Good
Kawata et al., 2016^20^	Retrospective cohort	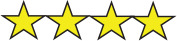	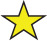	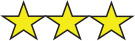	Good
Dell’Era et al., 2017^21^	Prospective cohort	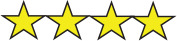	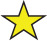	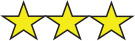	Good
Li et al., 2017^22^	Retrospective cohort	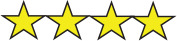	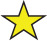	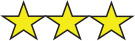	Good
Pillarisetti et al., 2017^23^	Retrospective cohort	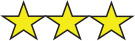	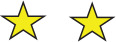	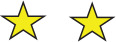	Good
Madeira et al., 2017^24^	Retrospective cohort	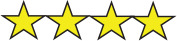	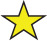	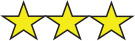	Good
Weng et al., 2017^25^	Retrospective cohort	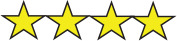	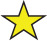	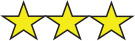	Good
Witt et al., 2018^26^	Retrospective cohort	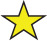	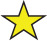	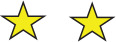	Poor
Narducci et al., 2018^27^	Retrospective cohort	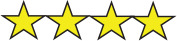	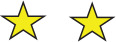	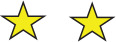	Good
Ruwald et al., 2019^28^	Prospective registry	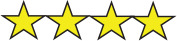	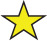	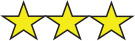	Good
Thomas et al., 2019^29^	Registry	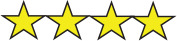		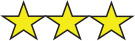	Poor
Theuns et al., 2021^30^	Retrospective cohort	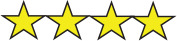	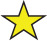	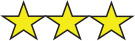	Good
Demarchi et al., 2021^31^	Retrospective cohort	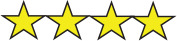	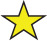	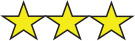	Good

*Abbreviation:* AHRQ, Agency for Healthcare Research and Quality.

**Table S2:** Covariate Analysis of Appropriate Implantable Cardioverter-defibrillator Shocks

	Univariate Analysis				Multivariate Analysis			
	ES (95% CI)	*Z* Value	*P* Value	*R*^2^ (%)	ES (95% CI)	*Z* Value	*P* Value	*R*^2^ (%)
Sample size	−0.001 (−0.005 to 0.002)	−0.77	.43	0				
Study design	0.59 (−0.16 to 1.33)	1.52	.12	12.9				
Follow-up (years)	0.09 (−0.14 to 0.32)	0.77	.44	0				
Mean age (years)	0.03 (−0.06 to 0.12)	0.65	.5	0				
Male	**0.04 (0.0007**–**0.08)**	**1.99**	**.04**	**28.2**	0.006 (−0.04 to 0.05)	0.26	.79	68.6
DM	0.009 (−0.03 to 0.04)	0.44	.65	0				
HTN	−0.004 (−0.02 to 0.02)	−0.34	.72	0				
CKD	−0.04 (−0.10 to 0.01)	−1.34	.17	15.5				
Mean BMI (kg/m^2^)								
Mean QRS (ms)	−0.02 (−0.07 to 0.02)	−1.09	.27	4.8				
AF	−0.007 (−0.04 to 0.02)	−0.4	.68	0				
ICM	0.01 (−0.01 to 0.04)	1.16	.24	6.2				
NYHA 3, 4	−0.003 (−0.03 to 0.02)	−0.27	.78	0				
Patients with improved LVEF	−0.01 (−0.05 to 0.02)	−0.8	.42	0				
Mean LVEF (%)	−0.01 (−0.12 to 0.08)	−0.3	.76	0				
CRTD	−**0.01 (**−**0.02 to** −**0.004)**	−**2.98**	**.002**	**48.3**	−0.01 (−0.02 to 0.001)	−1.76	.07	
ACEI/ARB	−0.01 (−0.08 to 0.06)	−0.31	.75	0				
BB	**0.06 (0.009**–**0.12)**	**2.29**	**.02**	**33.9**	**0.05 (0.01**–**0.1)**	**2.64**	**.008**	
MA	−0.01 (−0.04 to 0.01)	−0.99	.31	0				
Digoxin	0.02 (−0.04 to 0.09)	0.62	.53	0				
AAD	0.01 (−0.05 to 0.08)	0.42	.67	0				
Previous shocks	0.02 (−0.02 to 0.07)	0.88	.37	0				
Previous ATPs	0.002 (−0.01 to 0.02)	0.31	.75	0				

*Abbreviations:* AAD, anti-arrhythmic drugs; ACEI/ARB, angiotensin-converting enzyme inhibitor/angiotensin receptor blocker; AF, atrial fibrillation; BB, β-blocker; BMI, body mass index; CI, confidence interval; CKD, chronic kidney disease; CRT-D, cardiac resynchronization therapy-defibrillator; DM, diabetes mellitus; ES, effect size; HTN, hypertension; ICD, implantable cardioverter-defibrillator; ICM, ischemic cardiomyopathy; LVEF, left ventricular ejection fraction; MA, mineralocorticoid antagonist; NYHA, New York Heart Association.

**Table S3:** Covariate Analysis of Anti-tachycardia Pacing

	Univariate Analysis				Multivariate Analysis			
	ES (95% CI)	*Z* Value	*P* Value	*R*^2^ (%)	ES (95% CI)	*Z* Value	*P* Value	*R*^2^ (%)
Study design	0.19 (−0.57 to 0.96)	0.49	.62	0				
Follow-up (years)	**−0.18 (−0.35 to −0.007)**	**−2.04**	**.04**	34.15	−0.07 (−1.34 to 1.18)	−0.12	.9	10
Mean age in years	0.02 (−0.05 to 0.11)	0.65	.51	0				
Male	0.008 (−0.04 to 0.05)	0.32	.74	0				
DM	0.002 (−0.04 to 0.04)	0.09	.92	0				
HTN	−0.01 (−0.03 to 0.01)	−0.8	.42	0				
CKD	0.02 (−0.20 to 0.26)	0.23	.81	0				
Mean BMI	0.59 (−0.18 to 1.37)	1.49	.13	0				
Mean QRS	0.0004 (−0.05 to 0.05)	0.001	.99	0				
AF	0.01 (−0.01 to 0.04)	0.95	.34	0				
ICM	−0.01 (−0.04 to 0.01)	−0.94	.34	0				
NYHA 3, 4	**−0.03 (−0.06 to −0.003)**	**−2.19**	**.02**	51.81	−0.07 (−0.21 to 0.07)	−0.96	.33	
Patients with improved LVEF	0.01 (−0.01 to 0.05)	0.91	.35	0				
Mean LVEF	0.06 (−0.02 to 0.15)	1.44	.14	6.8				
CRTD	−0.004 (−0.03 to 0.02)	−0.29	.76	0				
ACE I/ARB I	0.04 (−0.01 to 0.10)	1.39	.16	17.8				
BB	0.01 (−0.10 to 0.13)	0.21	.83	0				
MA	−0.03 (−0.010 to 0.04)	0.03	.38	0				
Digoxin	−0.03 (−0.12 to 0.05)	−0.81	.41	0				
AAD	−0.07 (−0.23 to 008)	−0.9	.36	0				
Previous shocks	−0.03 (−0.12 to 0.05)	−0.75	.45	0				
Previous ATPs	−0.01 (−0.06 to 0.02)	−0.80	.42	0				

*Abbreviations:* AAD, anti-arrhythmic drugs; ACE/ARB, angiotensin-converting enzyme inhibitor/angiotensin receptor blocker; AF, atrial fibrillation; ATP, anti-tachycardia pacing; BB, β-blocker; BMI, body mass index; CKD, chronic kidney disease; CRT-D, cardiac resynchronization therapy-defibrillator; DM, diabetes mellitus; ES, effect size; HTN, hypertension; ICM, ischemic cardiomyopathy; LVEF, left ventricular ejection fraction; MA, mineralocorticoid antagonist; NYHA, New York Heart Association. Bolded values are statistically significant (*P* < 0.05).

**Table S4:** Covariate Analysis of Inappropriate Shocks

	Univariate Analysis				Multivariate Analysis			
	ES (95% CI)	*Z* Value	*P* Value	*R*^2^ (%)	ES (95% CI)	*Z* Value	*P* Value	*R*^2^ (%)
Sample	0.0006 (−0.0003 to 0.001)	1.33	.18	10				
Study design	−0.33 (−1.15 to 0.48)	−0.8	.42	0				
Follow-up (years)	0.01 (−0.20 to 0.22)	0.1	.91	0				
Mean age in years	0.06 (−0.008 to 0.13)	1.72	.08	20.6				
Male	−0.05 (−0.14 to 0.04)	−1.08	.27	0				
DM	0.03 (−0.007 to 0.07)	1.59	.11	14.5				
HTN	0.01 (−0.003 to 0.03)	1.65	.09	22				
CKD	**0.08 (0.04**–**0.12)**	**4.18**	**<.0001**	**100**	0.004 (−0.01 to 0.08)	0.31	.79	45.5
Mean BMI	0.11 (−0.15 to 0.39)	0.83	.4	0				
Mean QRS	0.004 (−0.03 to 0.04)	0.26	.78	0				
AF	0.02 (−0.01 to 0.05)	1.35	.17	10.8				
ICM	0.03 (−0.002 to 0.06)	1.84	.06	22.8				
NYHA 3, 4	−**0.02 (**−**0.05 to** −**0.0006)**	−**1.99**	**.04**	**52.4**	−0.001 (−0.02 to 0.04)	−1.76	.57	
Patients with improved LVEF	−0.01 (−0.04 to 0.01)	−0.91	.36	0				
Mean LVEF	0.001 (−0.11 to 0.11)	0.03	.97	0				
CRTD	−0.002 (−0.01 to 0.009)	−0.38	.7	0				
ACEI/ARB	−**0.04 (**−**0.08 to** −**0.007)**	−**2.32**	**.02**	**0**	−0.015 (−0.02 to 0.04)	−0.65	.15	
BB	−0.04 (−0.11 to 0.03)	−1.12	.26	8.3				
MA	0.001 (−0.05 to 0.06)	0.03	.23	0.96				
Digoxin	0.01 (−0.08 to 0.11)	0.32	.74	0				
AAD	−0.009 (−0.06 to 0.04)	−0.32	.74	0				
Previous shocks	**0.31 (0.02**–**0.59)**	**2.11**	**.03**	**88.2**	−0.01 (−0.05 to 0.03)	−0.56	.57	
Previous ATPs	−0.02 (−0.06 to 0.02)	−0.80	.42	0				

*Abbreviations:* AAD, anti-arrhythmic drugs; ACEI/ARB, angiotensin-converting enzyme inhibitor/angiotensin receptor blocker; AF, atrial fibrillation; ATP, anti-tachycardia pacing; BB, β-blocker; BMI, body mass index; CKD, chronic kidney disease; CRT-D, cardiac resynchronization therapy-defibrillator; DM, diabetes mellitus; HTN, hypertension; ICM, ischemic cardiomyopathy; LVEF, left ventricular ejection fraction; MA, mineralocorticoid antagonist; NYHA, New York Heart Association. Bolded values are statistically significant (*P* < 0.05).

**Table S5:** List of Procedure-related Complications in Different Studies

Study	Device Erosion	Device Malfunction	Device-pocket Hematoma	Pocket/Site Infection	Lead Infection	Lead Dislodgement	Lead Fracture	Overall Complication Rate
Looi et al., 2019^10^	—	—	1.6%	1.6%	—	—	6.6%	—
Arcinas et al., 2021^11^	0.5%	0.9%	0.9%	3.7%	1.4%	2.7%	2.7%	—
Yap et al., 2014^18^	0.4%	—	0.8%	2.3%	1.9%	7%	Yap et al., 2014^18^	0.4%
Weng et al., 2017^25^	—	—	—	—	—	—	—	5.1%

## References

[1] Friedman DJ, Parzynski CS, Varosy PD (2016). Trends and in-hospital outcomes associated with adoption of the subcutaneous implantable cardioverter defibrillator in the United States. JAMA Cardiol.

[2] Madhavan M, Waks JW, Friedman PA (2016). Outcomes after implantable cardioverter-defibrillator generator replacement for primary prevention of sudden cardiac death. Circ Arrhythm Electrophysiol.

[3] Tromp J, Ouwerkerk W, van Veldhuisen DJ (2022). A systematic review and network meta-analysis of pharmacological treatment of heart failure with reduced ejection fraction. JACC Heart Fail.

[4] Moss AJ, Schuger C, Beck CA (2012). MADIT-RIT trial investigators. Reduction in inappropriate therapy and mortality through ICD programming. N Engl J Med.

[5] Schrage B, Uijl A, Benson L (2019). Association between use of primary-prevention implantable cardioverter-defibrillators and mortality in patients with heart failure: a prospective propensity score-matched analysis from the Swedish heart failure registry. Circulation.

[6] Kini V, Soufi MK, Deo R (2014). Appropriateness of primary prevention implantable cardioverter-defibrillators at the time of generator replacement: are indications still met?. J Am Coll Cardiol.

[7] Yuyun MF, Erqou SA, Peralta AO (2021). Ongoing risk of ventricular arrhythmias and all-cause mortality at implantable cardioverter-defibrillator generator change: a systematic review and meta-analysis. Circ Arrhythm Electrophysiol.

[8] Bennett M, Parkash R, Nery P (2017). Canadian Cardiovascular Society/Canadian Heart Rhythm Society 2016 implantable cardioverter-defibrillator guidelines. Can J Cardiol.

[9] Poole JE, Gleva MJ, Mela T (2010). Complication rates associated with pacemaker or implantable cardioverter-defibrillator generator replacements and upgrade procedures: results from the REPLACE registry. Circulation.

[10] Looi KL, Gavin A, Cooper L, Dawson L, Slipper D, Lever N (2019). Outcomes of patients with heart failure after primary prevention ICD unit generator replacement. Heart Asia.

[11] Arcinas LA, Chew DS, Seifer CM (2021). Predictors of appropriate shock after generator replacement in patients with an implantable cardioverter-defibrillator. Pacing Clin Electrophysiol.

[12] Krahn AD, Lee DS, Birnie D (2011). Ontario ICD Database Investigators. Predictors of short-term complications after implantable cardioverter-defibrillator replacement: results from the Ontario ICD Database. Circ Arrhythm Electrophysiol.

[13] Liberati A, Altman DG, Tetzlaff J (2009). The PRISMA statement for reporting systematic reviews and meta-analyses of studies that evaluate healthcare interventions: explanation and elaboration. BMJ.

[14] Stang A (2010). Critical evaluation of the Newcastle-Ottawa scale for the assessment of the quality of nonrandomized studies in meta-analyses. Eur J Epidemiol.

[15] Schwarzer G, Rücker G (2022). Meta-analysis of proportions. Methods Mol Biol.

[16] Naksuk N, Saab A, Li JM (2013). Incidence of appropriate shock in implantable cardioverter-defibrillator patients with improved ejection fraction. J Card Fail.

[17] Sebag FA, Lellouche N, Chen Z (2014). Positive response to cardiac resynchronization therapy reduces arrhythmic events after elective generator change in patients with primary prevention CRT-D. J Cardiovasc Electrophysiol.

[18] Yap SC, Schaer BA, Bhagwandien RE (2014). Evaluation of the need of elective implantable cardioverter-defibrillator generator replacement in primary prevention patients without prior appropriate ICD therapy. Heart.

[19] House CM, Nguyen D, Thomas AJ, Nelson WB, Zhu DW (2016). Normalization of left ventricular ejection fraction and incidence of appropriate antitachycardia therapy in patients with implantable cardioverter defibrillator for primary prevention of sudden death. J Card Fail.

[20] Kawata H, Hirai T, Doukas D (2016). The occurrence of implantable cardioverter defibrillator therapies after generator replacement in patients who no longer meet primary prevention indications. J Cardiovasc Electrophysiol.

[21] Dell’Era G, Degiovanni A, Occhetta E (2017). Persistence of ICD indication at the time of replacement in patients with initial implant for primary prevention indication: effect on subsequent ICD therapies. Indian Pacing Electrophysiol J.

[22] Li X, Yang D, Kusumoto F (2017). Predictors and outcomes of cardiac resynchronization therapy extended to the second generator. Heart Rhythm.

[23] Pillarisetti J, Gopinathannair R, Haney MJ (2017). Risk of ventricular tachyarrhythmias following improvement of left ventricular ejection fraction in patients with implantable cardiac defibrillators implanted for primary prevention of sudden cardiac death. J Interv Card Electrophysiol.

[24] Madeira M, António N, Milner J (2017). Who still remains at risk of arrhythmic death at time of implantable cardioverter-defibrillator generator replacement?. Pacing Clin Electrophysiol.

[25] Weng W, Sapp J, Doucette S (2017). Benefit of implantable cardioverter-defibrillator generator replacement in a primary prevention population-based cohort. JACC Clin Electrophysiol.

[26] Witt CM, Waks JW, Mehta RA (2018). Risk of appropriate therapy and death before therapy after implantable cardioverter-defibrillator generator replacement. Circ Arrhythm Electrophysiol.

[27] Narducci ML, Biffi M, Ammendola E (2018). Appropriate implantable cardioverter-defibrillator interventions in cardiac resynchronization therapy-defibrillator (CRT-D) patients undergoing device replacement: time to downgrade from CRT-D to CRT-pacemaker? Insights from real-world clinical practice in the DECODE CRT-D analysis. Europace.

[28] Ruwald MH, Ruwald AC, Johansen JB (2019). Incidence of appropriate implantable cardioverter-defibrillator therapy and mortality after implantable cardioverter-defibrillator generator replacement: results from a real-world nationwide cohort. Europace.

[29] Thomas IC, Wang Y, See VY, Minges KE, Curtis JP, Hsu JC (2019). Outcomes following implantable cardioverter-defibrillator generator replacement in patients with recovered left ventricular systolic function: The National Cardiovascular Data Registry. Heart Rhythm.

[30] Theuns D, Niazi K, Schaer B, Sticherling C, Yap SC, Caliskan K (2021). Reassessment of clinical variables in cardiac resynchronization defibrillator patients at the time of first replacement: Death after replacement of CRT (DARC) score. J Cardiovasc Electrophysiol.

[31] Demarchi A, Cornara S, Sanzo A (2021). Incidence of ventricular arrhythmias and 1-year predictors of mortality in patients treated with implantable cardioverter-defibrillator undergoing generator replacement. J Am Heart Assoc.

[32] Herendael HV, Pinter A, Ahmad K, Korley V, Mangat I, Dorian P (2010). Role of antiarrhythmic drugs in patients with implantable cardioverter defibrillators. Europace.

[33] Kidwell GA, Greenspon AJ, Greenberg RM (1993). Use-dependent prolongation of ventricular tachycardia cycle length by type I antiarrhythmic drugs in humans. Circulation.

[34] Connolly SJ, Dorian P, Roberts RS (2006). Comparison of beta-blockers, amiodarone plus beta-blockers, or sotalol for prevention of shocks from implantable cardioverter defibrillators: the OPTIC study: a randomized trial. JAMA.

[35] Ruwald AC, Gislason GH, Vinther M (2018). Importance of beta-blocker dose in prevention of ventricular tachyarrhythmias, heart failure hospitalizations, and death in primary prevention implantable cardioverter-defibrillator recipients: a Danish nationwide cohort study. Europace.

[36] Migaj J, Kałużna-Oleksy M, Nessler J (2018). Impact of digoxin on risk of death in heart failure patients treated with b-blockers. Results from Polish part of ESC Heart Failure Long-Term Registry. Kardiol Pol.

[37] Merchant FM, Quest T, Leon AR, El-Chami MF (2016). Implantable cardioverter-defibrillators at end of battery life: opportunities for risk (re)-stratification in ICD recipients. J Am Coll Cardiol.

[38] Kramer DB, Buxton AE, Zimetbaum PJ (2012). Time for a change—a new approach to ICD replacement. N Engl J Med.

[39] Pillarisetti J, Reddy MY, Lakkireddy D (2014). Ejection fraction may improve but the scar still exists! The risk may be lower but not zero. J Am Coll Cardiol.

[40] Greenspon AJ, Prutkin JM, Sohail MR (2012). Timing of the most recent device procedure influences the clinical outcome of lead-associated endocarditis results of the MEDIC (Multicenter Electrophysiologic Device Infection Cohort). J Am Coll Cardiol.

[41] Bansal N, Szpiro A, Masoudi F (2017). Kidney function and appropriateness of device therapies in adults with implantable cardioverter defibrillators. Heart.

